# Enhancing Precision in Lumbar Spine Surgery Through Spinal Endoscopy: A Narrative Review with Expert Opinion

**DOI:** 10.3390/jpm16070365

**Published:** 2026-07-04

**Authors:** Bradley C. Nelson, Mark J. Lambrechts

**Affiliations:** Department of Orthopaedic Surgery, Washington University in St. Louis School of Medicine, St. Louis, MO 63110, USA; nelson.b@wustl.edu

**Keywords:** endoscopic, spine surgery, discectomy, unilateral laminectomy for bilateral decompression

## Abstract

Technological advances in spine surgery have allowed for significantly improved precision. Perhaps no technology has allowed for more personalized and precise surgery than endoscopic spine surgery. Although this technology has been around for decades, advancements in camera resolution have led to enhanced magnification and visualization of nerve root compression. Given our improved understanding of the interplay between spinal stability, spine pain, and muscle health, minimizing muscle disruption and bone resection has now become a key principle in spinal care. This narrative review will talk about common lumbar spine pathologies and how spinal endoscopy can be implemented to potentially improve patient care and outcomes.

## 1. Introduction

Lumbar spinal fusions are expected to increase over the next couple decades with the largest increases expected in the geriatric population [1]. As patients undergoing spine surgery become older, bone quality becomes less robust, leading to osteopenia and osteoporosis with resultant concerns for spinal fusion failure including adjacent segment disease and pseudarthrosis [2]. Preoperative optimization with anabolic bone agents, preoperative nutrition augmentation, and muscle strengthening may help reduce the risk of these complications, but utilization of alterative techniques to spinal fusion are becoming more realistic options [3].

Tubular and endoscopic spine surgery have become increasingly adopted by spine surgeons as technological advancements improve. While traditional spine surgery is “open”, which includes maximal muscle stripping, laminectomies, and partial or full facetectomies (which are destabilizing in nature), minimally invasive surgery (MIS) minimizes muscle disruption and bone resection.

Tubular surgery is performed through 2–3 cm incisions with muscle dilation and resection of muscle within the tube. Biportal endoscopy includes similar amounts of muscle stripping (albeit without muscle resection), while creating a cavity to allow for visualization of instruments and spinal anatomy. These can be thought of as nearly synonymous in terms of invasiveness, although clearly the incisions are smaller for the biportal endoscopy. Biportal endoscopy typically also offers significantly better visualization. Uniportal endoscopy is clearly the least invasive option with incisions as small as 7 mm and minimal need to disrupt any muscle. Long-term outcome data is still lacking regarding the benefit of these minimally invasive options, but there is clearly a theoretical benefit in minimizing the surgical footprint.

With the advancement of minimally invasive techniques in spine surgery, current treatment paradigms have shifted towards individualized, pathology-specific interventions. Targeted decompression, surgical corridor selection, and enhanced visualization of pathology using modern technology have significantly advanced spine care in today’s age. As lumbar surgery evolves, the clinical question is no longer whether minimally invasive approaches are preferable to open techniques, but rather which corridor and decompression strategy best suits a patient’s specific pathology, anatomy, instability profile, age, and bone quality. Although previous studies have compared various techniques in lumbar surgery, few have focused on how these approaches may be selected in a patient-specific manner to maximize decompression while minimizing tissue disruption and the necessity for spinal fusion. In this context, precision lumbar surgery refers to the individualized selection of approach and extent of decompression based on radiographic findings, symptom pattern, and patient-specific surgical risk.

This narrative review will discuss common lumbar conditions and surgical options available for patients who have not sustained adequate benefit from non-surgical interventions. The senior author will discuss his preference based on patient-specific symptoms and imaging findings seen on standard radiographs and magnetic resonance imaging (MRI) to personalize the approach to lumbar spine surgery and maximize precision. Therefore, the purpose of this review is to summarize the modern role of minimally invasive techniques across various common lumbar pathologies and to describe a practical, pathology-specific framework for selecting the least disruptive approach capable of achieving adequate decompression and symptom relief.

## 2. Methods

The manuscript is an expert opinion with a narrative review supported by selected literature and clinical examples. Therefore, a comprehensive systematic review of the literature on endoscopic and minimally invasive surgery was not performed. However, the authors utilized the PubMed database to access studies relevant to current techniques on spine surgery precision, evidence for current learning curves in spinal endoscopy, and to extract previous systematic reviews, database studies, and institutional studies comparing open versus tubular versus endoscopic surgery and their respective patient-reported outcomes and associated complications. To support the senior author’s clinical perspective on management of certain lumbar spinal pathology, relevant cases, including preoperative and postoperative imaging, were extracted from Epic to showcase representative clinical examples in utilizing precision spine surgery. Our institutional human resource protection office (HRPO) reviewed the manuscript and determined it does not involve activities subject to institutional review board (IRB) oversight, making the manuscript exempt from IRB approval.

## 3. Discussion

### 3.1. Lumbar Disc Herniations

There is clear, documented efficacy for surgical discectomy in patients who are unresponsive to conservative care. In fact, it is perhaps some of the highest quality data we have in spine surgery. Patients who underwent open lumbar discectomy sustained superior clinical outcomes versus conservative treatment for up to 8 years following surgery as described by the SPORT trial [4].

Although there has been increasing literature comparing open versus tubular versus endoscopic discectomy outcomes, meaningful clinical differences between MIS options have not been maintained across studies. A recent meta-analysis comparing open versus tubular versus biportal endoscopy found MIS discectomy (tubular and biportal endoscopy) had similar back pain, leg pain, and disability outcome scores at short-term and 12-month follow up, while open discectomy was associated with significantly worse back pain scores and Oswestry Disability Index (ODI) at short and 12-month postoperative time points [5].

A separate meta-analysis found biportal endoscopic patients had improved 2-week postoperative back pain and lower complication rates compared to tubular discectomy, although the study did include patients undergoing decompression without discectomy, which may provide some confounding [6]. Interestingly, an even earlier study comparing endoscopic discectomy to tubular and open discectomy found that open discectomy required longer hospital length of stay (LOS) and return to work time (RTW), while resulting in worse postoperative leg and back pain and ODI scores at final follow up. Hospital LOS, RTW, and patient-reported outcomes were similar between the endoscopic and tubular groups [7].

Aside from quicker recovery times, another major benefit to endoscopic spine surgery is improved access to foraminal and far lateral disc herniations. In open and tubular surgery, visualization of disc fragments is often limited, even after a Wiltse approach. True foraminal herniations can be even more difficult to fully visualize without facetectomies, which destabilize the spine and ultimately require fusion. Uniportal endoscopic discectomy (UED) and unilateral biportal endoscopic discectomy (UBED) overcome the limitations of tubular and open discectomies and allow placement of the camera in the foramen for annular defect and disc herniation visualization. For far lateral disc herniations, surgeons can dock in the foramen or dock extraforaminal, then “reverse” the camera to visualize and resect the extraforaminal disc herniation that would be otherwise challenging to treat without fusion. This extraforaminal docking technique, thus, provides excellent precision with an individualized approach to patient anatomy that was not accessible in the past.

It has been well documented that aggressive approaches to removal of disc herniations during discectomy procedures may result in accelerated disc degeneration due to aggressive disc debulking and an inability to visualize the annular defect [8]. Recent reports have advocated for the use of targeted sequestrectomy rather than radical or subtotal discectomy. A recent meta-analysis found sequestrectomy was associated with improved pain outcomes at 2 years with greater patient satisfaction, shorter operative times, and no increase in reherniation rate [9]. Additionally, endoscopic advancements in spine surgery have come with evolving management strategies for incidental durotomy. A 2019 retrospective study found that in 49 patients who experienced iatrogenic dural tears during endoscopic spine surgery, none required conversion to open surgery, with no worsening of surgical outcomes [10].

The downside to endoscopic approaches is the significant learning curve. While lumbar discectomies are usually some of the easier endoscopic surgeries to master [11], the learning curve is still much higher than open procedures with a pooled average of 20 UBED surgeries and 37.5 UED surgeries to become proficient [12]. However, this is likely the low end of the spectrum to become “safe and effective.”

More accurately, most surgeons should expect an 80% reduction in complications during endoscopic spine surgery for lumbar disc herniations after 40–70 cases, while achievement of an 80% reduction in leg pain occurs closer to 96 surgeries [13].

### 3.2. Expert’s Clinical Perspective

For primary disc herniations, the senior author has switched his practice entirely to UED. Full endoscopic techniques allow for visualization of any annular defect, and the disc herniation can be fully visualized. This allows for confidence that the entirety of the disc herniation has been resected. A sequestrectomy, as opposed to complete discectomy or subtotal discectomy, is performed to minimize long-term disc degeneration since the annular defect can be visualized and then utilized to remove free disc fragments as opposed to creation of a new annulotomy. This technique allows for enhanced precision care that has otherwise been inaccessible in spine surgery. UED also minimizes any muscle trauma, and it allows patients, including geriatric patients, to be discharged during the same day. Most importantly, primary foraminal and far lateral disc herniations are treated with discectomy instead of with spinal fusions. For far lateral disc herniations, the learning curve is quite high, and this should not be a surgeon’s first handful of cases [8]. The camera should be docked at the entrance to the foramen (or extraforaminal), and the camera can then be turned 180 degrees, so the exiting nerve root is visualized as it exits the foramen. This technique requires full visualization of the exiting nerve root to ensure adequate nerve root decompression.

**Figure 1:**
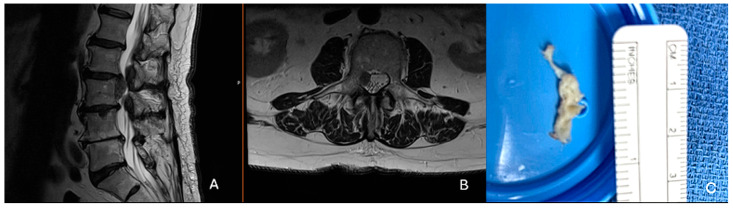
80-year-old male who is an ideal candidate for a right-sided L2–3 interlaminar endoscopic approach for a highly migrated disc herniation. (**A**): Preoperative T2-weighted sagittal MRI demonstrating the large, migrated disc herniation that has migrated to just rostral to the L3–4 disc space (**B**): Axial T2-weighted MRI at the mid L3 vertebrae (**C**): Picture of removed herniated disc fragment.

Although migrated disc herniations can be managed in a transforaminal fashion, the senior author has mostly switched towards utilizing interlaminar approaches as there is more versatility in accessing highly migrated herniations, which can be difficult to reach within the foraminal window (Figure 1). It also avoids contact and damage to the dorsal root ganglion, which can increase the likelihood of postoperative dysesthesias and radicular pain. Transforaminal techniques may be better options for broad-based disc herniations as they allow for a “wider” debulking corridor, without thecal sac manipulation and potential stretch injury to the cauda equina nerve roots. Revision lumbar discectomies provide the greatest challenge and should be approached by surgeons with significant endoscopic experience, especially if utilizing an interlaminar approach or the same approach as the prior surgeon. In these instances, preoperative computed tomography (CT) scans to assess adequate bone stock and planning the ideal docking location of the cannula is essential. Significant scar tissue will be encountered, and this will lead to longer operative times, greater fluid extravasation into soft tissues, and an increased risk of durotomy. An alternative option, especially when the disc herniation is rostral to L5–S1, is to perform a transforaminal approach to allow for normal soft tissue planes while accessing the disc herniation.

### 3.3. Lumbar Spinal Stenosis

Both uniportal and biportal endoscopic techniques are becoming more frequently utilized during lumbar laminectomies to facilitate recovery. A retrospective analysis found biportal endoscopy reduces postoperative pain and complication rates, largely driven by improved visualization and smaller incisions which minimizes operative times and reduces infections [14]. The ACS-NSQIP database also revealed that among patients undergoing single-level laminectomies, endoscopic surgery resulted in lower adverse outcomes than the open/tubular approaches (1.2% vs. 4.8%, *p* = 0.01) [15]. Among patients specifically undergoing full endoscopic unilateral laminectomy for bilateral decompression (ULBD) compared to tubular decompressions, a randomized controlled trial found similar patient reported outcomes, but shorter hospital LOS and lower blood loss following ULBD [16].

In general, unilateral laminectomies for bilateral decompression (in patients without severe spondylosis) are of relatively low case complexity [11]. However, resecting enough ligamentum flavum and medial facet joint can be time consuming if using a uniportal technique. Meanwhile, biportal techniques allow for improved visualization and a larger working portal (larger instruments), which comes at the expense of greater soft tissue stripping.

The role of fusion alongside decompression in lumbar spinal stenosis is a controversial topic. There is no intraoperative diagnostic threshold or strict consensus for “instability”, and as a result, surgeons often have different thresholds for fusion in combination with decompression. Ultimately, many physicians agree that fusion should be performed on patients who demonstrate clear instability on flexion–extension radiographs, but a shared-decision making model can be helpful when patients have severe back pain with significant facet arthropathy or degenerative disc disease that may be contributing to axial back pain [17].

### 3.4. Expert’s Clinical Perspective

There is still conflicting data on the superiority of individual decompression techniques, especially for multi-level decompressions. If more than a two-level decompression is required, either biportal endoscopic, tubular or open techniques can be considered given the current limitations available for uniportal techniques. The decision will come down to surgeon personal preference based on their comfort level with each technique.

**Figure 2:**
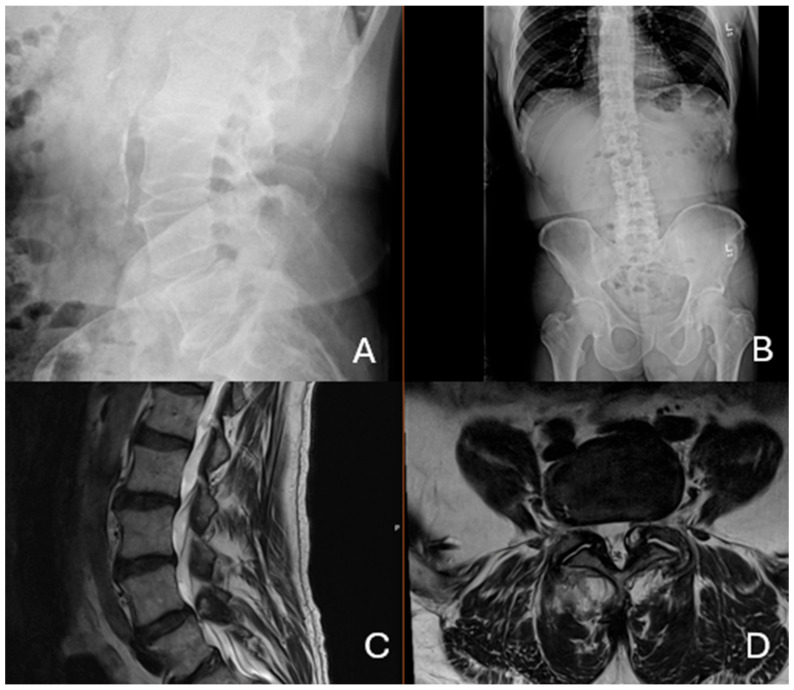
75-year-old male with radiating left leg pain with a synovial facet cyst but without any instability. (**A**): Preoperative lateral radiograph demonstrating normal sagittal alignment. (**B**): Preoperative AP radiograph demonstrating normal coronal alignment. (**C**): Preoperative T2-weight sagittal MRI demonstrating a left-sided synovial facet cyst (**D**): Preoperative T2-weighted axial MRI demonstrating impingement of the left L5 traversing nerve root due to a compressive synovial facet cyst.

The senior author still performs open decompressions for cases of severe stenosis without instability, especially when there are multiple levels of severe canal stenosis. In patients with synovial facet cysts causing severe central canal stenosis, or lateral recess stenosis, uniportal ULBD with cyst resection minimizes instability and muscle disruption. An example of a straightforward synovial facet cyst resection with an endoscope is seen in Figure 2. Iatrogenic instability is highly uncommon in endoscopic approaches as the contralateral facet is often left relatively undisrupted. The senior author has never converted these procedures to fusion due to overly aggressive resection causing instability. The senior author reserves preoperative planning of fusions for patients with instability on flexion–extension imaging in the setting of additional large facet joint effusions, indicating a high probability of synovial facet cyst recurrence. The continuous saline irrigation allows for easier plane development between the synovial facet cyst and the thecal sac and reduces the risk of dural disruption. However, it should be noted that some cysts have already fully eroded the dura, and an inlay collagen patch with a dural sealant, or an inlay and overlay collagen patch with dural sealant, may be required if a defect greater than a millimeter is observed [18].

### 3.5. Spondylolisthesis

Numerous high-quality studies have discussed the merit of decompression alone versus decompression and fusion in the setting of lumbar spinal stenosis with spondylolisthesis. In general, older patients are more likely to undergo lumbar decompression without fusion, while younger patients are more likely to undergo decompression and spinal fusion, with a meta-analysis suggesting that decompression minimizes complications at the risk of a higher revision rate [19]. In fact, this is the main finding of the NORDSTEN trial, which randomized patients to decompression alone versus decompression and fusion with results indicating decompression is non-inferior at two years, but with a greater risk of revision [20]. Interestingly, a surgeon’s recommendation does not impart greater outcomes when comparing the two surgical options, indicating a continued lack of understanding regarding which patients should undergo a decompression alone versus which patients are at high-risk of improvement failure following a simple decompression [21].

Since surgeons often perform fusion after the lumbar decompression due to preoperative instability (and heighted postoperative instability after muscle disruption and lamina and facet resection), limiting muscle trauma and bony resection may further limit additional instability. While long-term studies comparing open versus endoscopic decompressions in patients with spondylolisthesis are not available, short-term studies indicate lower reoperation rates and conversions to fusion compared to open decompression surgery [22]. Even amongst stable grade II and III spondylolistheses, endoscopic decompressions show favourable short-term outcomes, with significant improvement in symptoms without slip progression [23]. A separate retrospective study in a slightly younger patient population found patients undergoing endoscopic decompression had only a 7% slip progression at one-year minimum follow up and significant symptomatic improvement [24].

### 3.6. Expert’s Clinical Perspective

**Figure 3:**
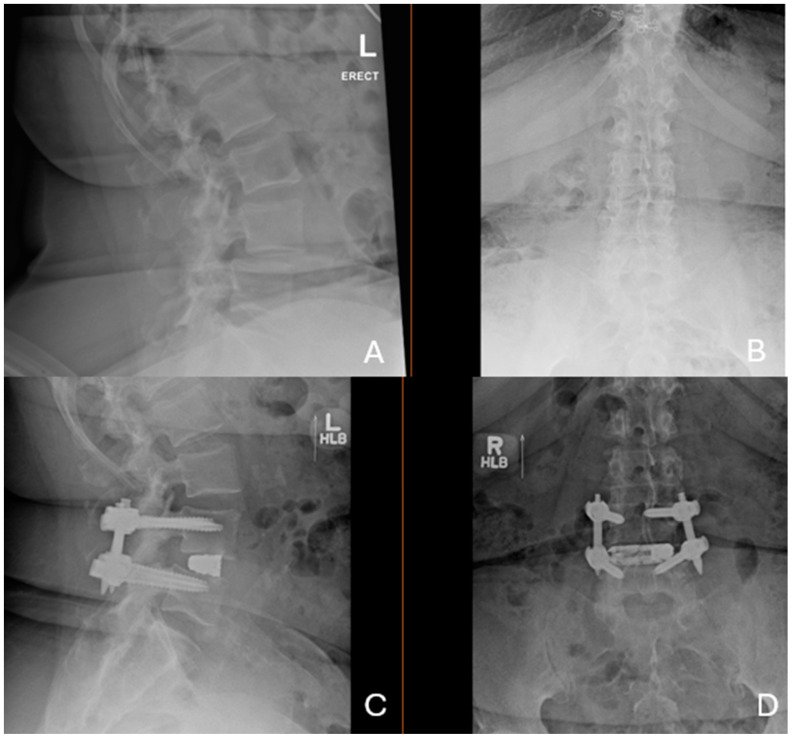
68-year-old female with a mobile spondylolisthesis and radiating leg pain. (**A**): Preoperative lateral radiograph demonstrating an L4–5 spondylolisthesis. (**B**): Preoperative AP radiography demonstrating normal coronal alignment. (**C**): 6-month lateral postoperative radiograph after a minimally invasive transforaminal lumbar interbody fusion with posterior instrumentation and fusion demonstrating complete spondylolisthesis reduction with disc height restoration. (**D**): 6-month postoperative AP radiograph.

**Figure 4:**
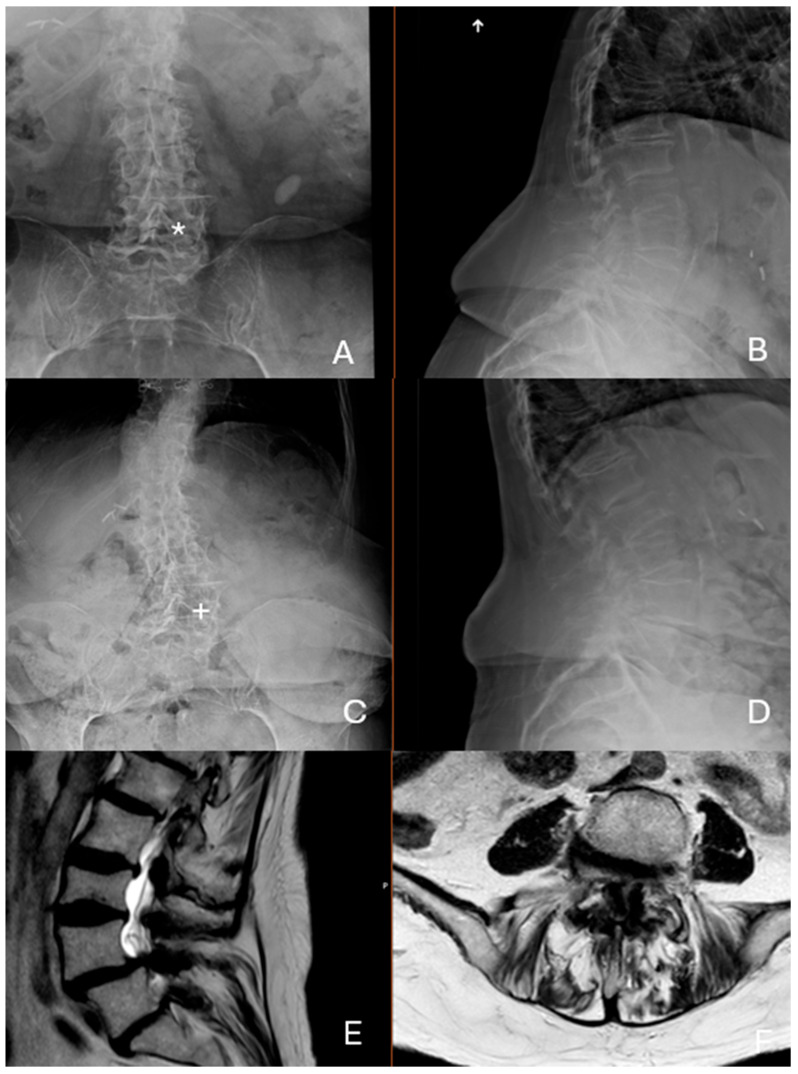
An 85-year-old female with L4–5 spondylolisthesis, severe central canal stenosis, and lost ability to ambulate due to pain severity. (**A**): Preoperative AP radiograph demonstrating lumbar scoliosis and severe osteoporosis with a small left sided interlaminar window (*). (**B**): Preoperative lateral radiograph showing an L4–5 spondylolisthesis. (**C**): 6-month postoperative radiograph demonstrating the left side bony resection (+) needed for the unilateral laminectomy with bilateral decompression. (**D**): Lateral radiograph demonstrates there is no slip progression at 6 months. (**E**): Preoperative T2-weighted sagittal MRI showing the L4–5 spondylolisthesis and severe central canal stenosis. (**F**): Preoperative T2-weighted axial MRI demonstrates severe central canal stenosis.

For younger patients with spondylolisthesis and instability, the senior author performs fusion with attempted reduction and improvement in segmental alignment goals, when necessary (Figure 3). In elderly patients, especially those with static spondylolisthesis, the senior author performs uniportal ULBD to minimize muscle disruption and bony resection while maximizing central canal decompression (Figure 4) [25]. These can be challenging cases due to a narrow interlaminar window and large amounts of facet spondylosis. Surgeons are recommended to have experience prior to performing these surgeries as it requires ventral spinous process resection (an over-the-top decompression) to create enough access for complete ligamentum flavum resection and adequate contralateral nerve root decompression. Durotomies and nerve root injuries can be consequences of insufficient bony work prior to ligament resection.

Given the prior literature available and the senior author’s own prior experiences, the risk of adverse complications is much lower with endoscopic central canal decompression and leads to very high patient satisfaction rates with a relatively low complication profile in comparison to decompression with fusion [22]. However, patients with foraminal stenosis and predominantly exiting nerve root pain are much less likely to have improvement in their symptoms when undergoing foraminoplasty and disc extrusion resection. In the senior author’s practice, severe vertical foraminal stenosis, especially when predominantly due to pedicle and endplate entrapment of the nerve, is an indication for more aggressive decompression with fusion [26].

### 3.7. Scoliosis

There is limited high quality evidence evaluating the efficacy and durability of decompression surgery in patients with scoliosis. This is due to high variability in curve magnitude and location of compression (e.g., central canal stenosis versus foraminal stenosis in the curve concavity). The limited data that currently exists suggests that decompression alone may have favourable outcomes with relatively low risk of reoperation rate and curve progression [24]. Similar findings were found in a propensity-matched retrospective study when comparing minimally invasive short-segment fusions versus minimally invasive decompression in patients with lumbar scoliosis. There were minimal differences in VAS-leg, VAS-back, and Oswestry Disability Index (ODI) with similar minimal clinically important differences (MCID) between groups [27]. While some data does support the efficacy of transforaminal endoscopic decompression (for foraminal stenosis) versus limited fusion within lumbar scoliosis curves, data is limited to small samples [27]. If isolated decompressions are performed, data appears to indicate that coronal cobb angles should be limited to less than 20 degrees as short segment fusions appear superior to decompressions alone when the cobb angles are greater than 20 degrees [28]. In a small retrospective study evaluating transforaminal endoscopic decompression versus fusion, the authors found only an average of 1.5 degrees of curve progression in the endoscopic treatment group, but the curves were limited to an average of about 20 degrees in the decompression group as compared to almost 40 degrees in the limited fusion group [29].

### 3.8. Expert’s Clinical Perspective

Like patients with spondylolisthesis, scoliosis patients with central canal stenosis can be effectively treated with ULBD [30]. When patients present with neurogenic claudication and/or lumbar radiculopathy due to central canal stenosis, the senior author’s preferred treatment is uniportal ULBD to minimize curve progression. The senior author attempts to minimize facet resection, and if the facet appears to be highly migrated into the midline of the canal open treatment, can minimize the risk of durotomy.

In most instances, transforaminal decompression is avoided due to the concern that partial facet and/or pedicle resection may worsen curve progression. For foraminal stenosis cases, decompression with fusion is indicated. To determine whether a partial or complete scoliosis fusion is necessary, utilization of radiographs and patient symptoms (magnitude of back pain versus leg pain and overall coronal and sagittal alignment on scoliosis films) help determine if a limited fusion or a fusion with scoliosis correction is indicated. In these cases, endoscopic surgery allows for precise decompression and increases treatment options outside of historical standards of care of fusion by minimizing muscle damage and bony resection.

### 3.9. Practical Applications and Future Directions

The application of this review lies within advocating for pathology-specific surgical planning rather than a one-size-fits-all approach. The concepts and perspectives summarized here may help surgeons identify patients in whom targeted and intentional decompression can provide adequate relief while minimizing post- and perioperative morbidity. This may be especially important in older adults, medically frail patients, and patients with poor bone quality. Additionally, this framework may assist with preoperative counselling by clarifying when fusion remains necessary and when technical complexity or patient-specific factors, such as instability, should necessitate a different approach. These principles may also help guide training by identifying pathology more suitable for those early in endoscopic training and which should be reserved for surgeons with greater procedural volume.

Future research should focus on prospective studies with longer follow-up comparing pathology-specific approaches to better define when endoscopic decompression alone is sufficient and when fusion remains necessary. Important priorities include long-term assessment of reoperation rates, adjacent-segment disease, learning curve progression, and patient-reported outcomes. Additional research is also needed to standardize definitions of lumbar instability and to better characterize the learning curve across different endoscopic techniques. Lastly, studies evaluating cost-effectiveness and outcomes after pathology-specific approaches for geriatric populations may further define the role of precision lumbar surgery in spine care.

This review has several limitations. First, it is a narrative review and therefore subject to selection bias in article inclusion. Second, portions of the discussion incorporate the senior author’s expert clinical preference which may not be generalizable across institutions or training backgrounds. This should be considered when interpreting this proposed change in paradigm.

## 4. Conclusions

Endoscopic spine surgery comes with new opportunities to perform precision lumbar spine surgery, as the area of pathology can be targeted with minimal collateral soft tissue destruction. However, biportal, and especially uniportal endoscopy, have very steep learning curve with true mastery (including minimizing complications while maximizing patient improvement) potentially requiring 100 surgeries. As technology continues to improve, individualized approach selection and anatomy-specific targeting with endoscopic visualization will continue to enhance precision spine care.

## Data Availability

No new data were created or analyzed in this study. The use of AI was not utilized for manuscript preparation or editing.

## Abbreviations

The following abbreviations are used in this manuscript:

MIS	minimally invasive surgery
MRI	Magnetic resonance imaging
ODI	Oswestry disability index
LOS	Length of stay
RTW	Return to work
UED	Uniportal endoscopic discectomy
UBED	Unilateral biportal endoscopic discectomy
CT	Computed tomography
ULBD	Unilateral laminectomy for bilateral decompression
IRB	Institutional Review Board
HRPO	Human Research Protection Office
